# Natural Biofortification in Semi-Natural Mountain Meadows: Ecological Drivers of Forage Trace Mineral Profiles

**DOI:** 10.3390/plants15213399

**Published:** 2026-11-02

**Authors:** Adrián Jarne, Asunción Usón, Ramón Reiné

**Affiliations:** Departamento de Ciencias Agrarias y del Medio Natural, Escuela Politécnica Superior, Universidad de Zaragoza, Ctra Cuarte s/n, 22071 Huesca, Spain; mauson@unizar.es (A.U.); rreine@unizar.es (R.R.)

**Keywords:** biodiversity, micronutrients, Pyrenean meadows, species functional groups, ICP-OES, forage quality, Ash content, Structural equation modelling (SEM)

## Abstract

Trace mineral dynamics in mountain pastures remain poorly quantified. This study evaluates the ecological drivers of forage micronutrient profiles (Fe, Cu, Mn, Zn) across 12 Pyrenean hay meadows. Over 3 years, 212 forage samples were analysed for botanical composition, standing biomass, and mineral content. Data were evaluated using PCA, Structural Equation Modelling (SEM), and tertile abundance partitioning. Subclinical Mn (47.5 ± 39.2 mg kg^−1^ DM) and Zn (22.4 ± 9.3 mg kg^−1^ DM) deficiencies were widespread. Non-leguminous forb cover drove Zn (R^2^ = 0.550, *p* < 0.001) and Cu (R^2^ = 0.428, *p* < 0.001) enrichment, reaching a target functional threshold at 20% to 25% cover without reducing biomass yield. Conversely, grass-dominated swards increased total ash via silica rather than trace minerals. Mn dynamics responded to thermal time, whereas Fe depended largely on soil contamination. Maintaining a 20% to 25% forbs cover target offers a practical, low-cost strategy to secure trace element self-sufficiency in extensive mountain livestock systems.

## 1. Introduction

Semi-natural grasslands are among the most biodiverse ecosystems in Europe and provide essential ecosystem services, including carbon sequestration, soil protection, and the maintenance of cultural landscapes [1,2]. In mountain regions such as the Aragonese Pyrenees, these grasslands support extensive livestock systems, where hay meadows constitute the main source of winter forage [3,4]. Under the low-input conditions typical of these systems, forage quality is a critical determinant of animal health, productivity, and farm economic viability [5,6]. While forage quality has traditionally been evaluated based on yield, energy content, and crude protein, increasing attention is being paid to the role of trace minerals in ruminant nutrition.

Iron (Fe), copper (Cu), manganese (Mn), and zinc (Zn) are among the most relevant trace elements for livestock health [7,8,9]. Copper and Zn deficiencies are widely reported in grassland forages across temperate regions and are associated with reduced growth, impaired immune function, and reproductive disorders, often without clear clinical symptoms [10,11]. Manganese generally shows intermediate adequacy, whereas Fe is rarely below recommended dietary allowances and frequently occurs at concentrations exceeding animal requirements [12]. Excess Fe intake is of particular concern because of its antagonistic effect on Cu absorption, potentially inducing functional Cu dietary insufficiency even when forage Cu concentrations appear adequate [7]. These interactions are especially relevant in mountain meadows, where soil characteristics and delayed cutting dates may favour high Fe availability and reduced Cu bioavailability.

Conventionally, trace element deficiencies are mitigated via exogenous supplementation [13]. However, in low-input mountain systems, purchasing and distributing commercial minerals creates a substantial economic burden for extensive farms [14]. Operationally, free-choice supplements often result in uneven individual intake, leaving some animals subclinically deficient, and fail to resolve dietary antagonisms such as soil-derived Fe blockage [15]. In contrast, natural biofortification through sward management offers a low-cost, sustainable strategy to enhance intrinsic micronutrient concentration and secure livestock self-sufficiency [16,17]. In the context of semi-natural grassland systems, ‘natural biofortification’ (or botanical biofortification) does not imply agronomic mineral fertilization or genetic modification. Rather, it refers to the strategic management of sward functional composition, specifically promoting native mineral-accumulating species such as non-leguminous forbs, to naturally elevate intrinsic forage micronutrient concentration and achieve livestock self-sufficiency.

Plant biodiversity has been proposed as an important driver of forage quality within the Biodiversity–Ecosystem Functioning (BEF) framework [18,19]. Species-rich plant communities may enhance nutrient acquisition through niche complementarity, differences in rooting depth, and variation in phenology and nutrient uptake strategies [20,21]. In contrast, intensively managed grasslands dominated by a small number of highly productive grass species often exhibit a dilution effect, whereby rapid biomass accumulation leads to reduced mineral concentrations in herbage. From this perspective, diverse grasslands could potentially provide forage with higher trace mineral density [22,23].

However, species richness alone has proven to be an inconsistent predictor of forage mineral composition. Increasing evidence indicates that functional group composition—particularly the relative contribution of grasses, legumes, and non-leguminous forbs—is a more reliable determinant [24,25]. Numerous studies have shown that legumes and forbs generally contain higher concentrations of Cu, Zn, and Mn than grasses and are therefore often described as mineral accumulators [23,26,27]. In contrast, grasses typically produce greater biomass but with lower micronutrient concentration. Deep-rooted non-leguminous forbs such as chicory (*Cichorium intybus*) and plantain (*Plantago lanceolata*), as well as common species in mountain meadows (e.g., *Taraxacum officinale* and *Sanguisorba minor*), have repeatedly been identified as particularly rich in trace minerals [27,28].

Management practices exert a strong influence on functional group composition and, consequently, on forage mineral profiles [29,30]. Nitrogen fertilization, early and frequent cutting, and high management intensity tend to promote competitive grasses at the expense of legumes and non-leguminous forbs, increasing yield but often reducing forage micronutrient concentrations [31]. In contrast, extensive management typical of mountain meadows—characterized by low or no nitrogen inputs, one or two annual cuts, and aftermath grazing—generally maintains a higher proportion of legumes and non-leguminous forbs, potentially enhancing mineral supply to livestock [32,33].

Phenological stage at harvest further modulates these patterns. As plants mature, increasing stem proportion and structural tissue development are typically associated with declining trace mineral concentrations, particularly in grasses [8,25]. In mountain environments, cutting is often delayed due to climatic constraints and traditional management calendars, which may exacerbate mineral dilution in grass-dominated swards [5]. However, swards with a higher proportion of non-leguminous forbs and legumes may partially buffer these declines, as these species functional groups often maintain relatively higher mineral concentrations later into the growing season [23,27].

Despite the recognized importance of botanical composition for forage quality, quantitative information linking plant diversity and functional group composition to trace mineral concentrations in semi-natural mountain meadows remains limited. Few studies have examined whether identifiable ecological thresholds—such as minimum levels of species richness or functional group cover—exist beyond which forage mineral density increases significantly. Moreover, most available studies focus on individual species [27] or experimental mixtures [20,34], whereas data from semi-natural meadows spanning real management and environmental gradients are scarce [33,35].

This knowledge gap has important practical implications for mountain livestock systems, where Cu and Zn supplementation is commonly required, while Fe supplementation is rarely necessary and may even be detrimental [7]. Improving the intrinsic mineral quality of hay through vegetation management could reduce reliance on external mineral inputs, enhance animal health, and increase the multifunctional value of semi-natural grasslands [1,2]. The objective of this study is therefore to evaluate the influence of botanical diversity and functional group composition on the concentrations of Fe, Cu, Mn, and Zn in semi-natural hay meadows across a combined management and altitudinal gradient in the Aragonese Pyrenees. Using multivariate ordination, structural equation modelling, and a categorical approach based on tertiles, we aim to identify the functional groups driving trace mineral dynamics and to determine whether a non-linear saturation threshold in non-leguminous forb cover optimizes forage micronutrient concentration without compromising standing biomass yield.

## 2. Results

### 2.1. Baseline Ecological Characterization and Comparison with Nutritional Baselines

The evaluation of the 12 semi-natural mountain meadows across the Aragonese Pyrenees captured a broad structural, botanical, and nutritional gradient. Sward composition, general quality matrices, and trace element profiles displayed substantial livestock-nutrition variations (Table 1).

The mountain forage exhibited high baseline values for plant biodiversity, with species richness averaging over 33 species per plot. In terms of macro-nutritional indicators, total ash content (7.14% ± 1.46%) sat slightly below standard lowland baselines [32], highlighting the distinct low-input mineral composition of these high-altitude ecosystems. Regarding micronutrient constraints for ruminants, both Manganese (47.54 mg kg^−1^) and Zinc (22.44 mg kg^−1^) contents fell beneath the optimal nutritional thresholds advised in international literature, signifying a latent risk of trace element dietary insufficiency.

Conversely, mean copper concentrations (10.64 mg kg^−1^ ± 19.28 mg kg^−1^) appeared generally adequate compared to global reference baselines. However, the standard deviation exceeding the mean reflects a strongly right-skewed distribution, driven by localized hotspots of high Cu accumulation in specific forb-rich swards. This pronounced distributional asymmetry further justifies our use of non-parametric analytical approaches (Spearman rank correlation, Kruskal–Wallis tests, and tertile partitioning) throughout the study. Meanwhile, Iron distributions showcased a high variability (154.25 ± 181.14 mg kg^−1^) frequently breaking the upper limits of physiological requirement and threatening trace cation absorption through potential mineral antagonisms.

### 2.2. Integrated Bivariate Pathways: Sward Architecture and Total Ash as Drivers of Trace Element Density

To outline the linear trajectories linking sward botanical traits, mineral concentration, and individual micronutrient deliveries, Spearman’s rank correlation coefficients (ρ) were unzipped. To maximize analytical compactness and avoid graphical duplication, botanical configurations and Total Forage Ash (%) were coupled into a single master correlation network (Table 2).

The correlation analysis revealed that botanical diversity and functional group identity significantly correlated the micronutrient content of the forage (Table 2). Species richness emerged as a consistent positive driver for most minerals, showing highly significant correlations with Mn (ρ = 0.39, *p* < 0.001), Zn (ρ = 0.34, *p* < 0.001) and Cu (ρ = 0.23, *p* < 0.001). This suggests that more diverse swards are associated with higher overall mineral density. Similarly, the cover of other families (non-graminoid/non-leguminous) exhibited the strongest positive association with Zn (ρ = 0.40, *p* < 0.001) and Cu (ρ = 0.29, *p* < 0.001), highlighting the role of these less common species as mineral accumulators.

In contrast, grass cover showed a distinct inhibitory effect, displaying significant negative correlations with Zn (ρ = −0.20, *p* < 0.01) and Cu (ρ = −0.14, *p* < 0.05). This indicates that swards dominated by grasses tend to have lower concentrations of these specific micronutrients. Legume cover contributed positively and significantly only to Mn levels (ρ = 0.33, *p* < 0.001), while showing no significant influence on other minerals.

Notably, iron (Fe) concentrations appeared to be independent of botanical composition, with no significant correlations observed across any of the studied variables. This general overview provided by the Spearman coefficients is further detailed by the categorical analysis presented in the following sections.

### 2.3. Non-Linear Structural Thresholds Across Sward Abundance Gradients

#### 2.3.1. Threshold Effects and Partitions in Forage Ash Dynamics

To isolate distinct ecological boundaries and identify potential non-linear shifts in the general mineral template, total forage ash content (%) was partitioned across the discrete abundance tertiles (Low, Medium, and High) of the primary botanical and diversity indicators (Figure 1). Global inference via the non-parametric Kruskal–Wallis test confirmed that sward organizational architecture significantly modulated gross ash accumulation, displaying both step-wise and threshold-dependent behaviours.

Legumes Cover (%) established itself as the most consistent and powerful negative driver of gross ash dynamics (*p* = 0.0010). Dunn’s post-hoc analysis validated a strict, stepwise statistical separation among all three abundance tiers; median forage ash percentages progressively dropped from the Low abundance group (a) to the Medium group (b), minimizing definitively within the High legume canopy (c).

Species Richness similarly exerted a highly significant downward pressure on the forage ash baseline (*p* = 0.0041) but operated via a discrete threshold mechanism rather than a continuous trilinear decline. Pairwise comparisons revealed that the transition from a Low to a Medium richness canopy did not induce structural modifications in the mineral matrix (both sharing the b marker). However, when taxonomic diversity breached the High richness threshold, a sharp, isolated statistical compression of total ash values was triggered (a), demonstrating that a high critical baseline of plant diversity is mandatory to alter the macro-mineral profile of the sward.

Conversely, Grass Cover (%) actively forced the mineral matrix upward (*p* = 0.0468). This positive relationship was characterized by a strict boundary contrast; significant structural differences were restricted to the extreme distribution margins, where the High grass dominance tertile (b) significantly outpaced the Low grass baseline (a). Finally, the abundance of Other Families Cover (%) (non-leguminous forbs) remained entirely decoupled from these structural dynamics (*p* = 0.8517, displaying overlapping, homogeneous ash distributions across all abundance categories (a).

#### 2.3.2. Micronutrient Concentration Profiles and Saturation Thresholds Across Tertiles

To evaluate how sward structural modifications translate into individual trace element deliveries, the concentrations of individual micronutrients (Fe, Cu, Mn, and Zn) were tracked across the same abundance tertile frameworks (Figure 2). Non-parametric testing unzipped highly divergent accumulation boundaries across the different metals.

Manganese (Mn) concentrations exhibited a consistent and significant stepwise increase (*p* < 0.001) across all positive botanical gradients. The transition from Low to High abundance tertiles in both Species Richness and Legumes Cover resulted in a distinct statistical separation of the means, reaching maximum values in the highest tertiles. Similarly, Zinc (Zn) levels were significantly higher in swards with greater species diversity and “Other Families” cover. In the latter, a saturation effect was noted, where both Medium and High abundance groups significantly outperformed the Low group (*p* < 0.001) but did not differ from each other.

In contrast, Copper (Cu) concentrations responded more selectively, with significant differences confirmed primarily between the extreme tertiles (Low vs. High) in Species Richness and Other Families cover. The impact of Grass Cover reinforced the negative trend observed in the correlation analysis; as grass dominance increased, a significant reduction in Mn and Zn concentrations was recorded, with the High abundance tertile showing the lowest mineral density. Finally, Iron (Fe) concentrations remained stable across all botanical gradients, with no significant differences between tertiles, indicating that Fe levels are independent of the sward’s botanical composition in the studied system.

### 2.4. Multivariate Space and Trait Trade-Offs: Unsupervised PCA

To synthesize the multi-trait trade-offs, environmental constraints, and nutritional outcomes within an unsupervised multivariate environment, a Principal Component Analysis (PCA) was deployed. The first two principal components accounted for 46.8% of the collective dataset variance, with PC1 and PC2 describing 27.6% and 19.2% of the variance, respectively (Figure 3).

To further isolate the true multivariate structure of trace minerals, a restricted Principal Component Analysis (PCA) was executed using exclusively the four micronutrient concentrations (Fe, Cu, Mn, and Zn) as active variables (n = 212). The first two principal components accounted for a cumulative explained variance of 65.1%, with PC1 explaining 40.4% and PC2 explaining 24.7% of the total variance (Figure 3).

The factor loadings (Table 3) revealed a highly specialized compartmentalization of the micronutrients. PC1 was heavily dominated by the cooperative variance of Zinc (loading = 0.687) and Copper (loading = 0.656). This horizontal axis effectively defines a metabolic biofortification gradient, confirming that Cu and Zn concentrations covary synergistically within the pasture ecosystem. In contrast, Iron (0.198) and Manganese (0.242) exerted minor influences on this primary axis.

The vertical axis (PC2) captured a stark, orthogonal divergence between Iron and Manganese. Fe displayed a dominant positive loading on PC2 (0.809), whereas Mn exhibited a strong negative loading on the same axis (−0.587). This geometrical opposition proves that Fe and Mn dynamics operate under entirely independent regulatory mechanisms, both from each other and from the Cu-Zn core matrix.

The most compelling methodological validation emerged from mapping Ash content (%) as a continuous colour gradient over the sample points. Samples characterized by high ash content (represented by the yellow hues in image_997817.png) clustered systematically and exclusively in the upper quadrant of the biplot, tracking the exact positive trajectory of the Fe vector (loading = 0.809). Conversely, low-ash samples (blue hues) clustered toward the lower and central regions. Because ash content was treated as a supplementary environmental variable and did not contribute to the mathematical construction of the PCA space, its definitive alignment with the Fe vector provides objective, uncoupled evidence that the total mineral ash fraction in the hay directly influences the inflation of Fe levels, corroborating the exogenous soil contamination pathway.

### 2.5. Hierarchical Structural Pathways and Direct Drivers of Micronutrient Concentrations

To disentangle the complex networks of direct and indirect influences regulating trace element concentrations, multivariate ordinary least squares (OLS) structural regression pathways were computed individually for each micronutrient. This causal framework successfully quantified the specific weight (β) of sward architectures, forage quality mediators, and agro-climatic drivers, explaining a substantial proportion of trace mineral variance across the ecosystem (Table 4).

#### Specific Pathway Configurations for Individual Microelements

The structural pathways mapped a highly specialized control hierarchy for each trace metal across the pasture ecosystem (Figure 4):

Zinc Pathway (R^2^ = 0.550): Zinc dynamics represented the most predictable system in our study, with over 55% of its variance successfully coordinated by the structural model. The percentage cover of Other Families (non-leguminous forbs) exerted a dominant, highly significant positive direct pathway (β = 18.421, *p* < 0.001), confirming that forb abundance serves as the primary biological key contributor driving Zn concentration. Gross mineral accumulation via Total Forage Ash (%) acted as a secondary reinforcing positive pathway (β = 14.215, *p* < 0.001). After controlling for these parameters, direct negative impacts from grass dominance or yield dilution were rendered non-significant (*p* > 0.05), proving their effects are fully mediated by sward architecture.

Copper Pathway (R^2^ = 0.428): Copper accumulation was driven by a multi-tiered synergy between biology and quality matrices. Other Families Cover (%) (β = 9.541, *p* < 0.001) and Total Forage Ash (%) (β = 11.204, *p* < 0.001) operated as critical positive structural pathways. Crucially, Crude Protein (%) displayed a robust, independent positive causal path (β = 6.412, *p* = 0.003), indicating that nitrogen-dense vegetative structures directly support higher Cu concentrations in the forage.

Manganese Pathway (R^2^ = 0.312): Manganese status broke away from the macro-mineral matrix, showing a zero-causal relationship with total ash (*p* = 0.913). Instead, Mn was governed by a powerful combination of temporal and climatic factors; Cutting Date (DOY) (β = 22.145, *p* < 0.001) and Growing Degree-Days (β = 12.411, *p* < 0.001) functioned as the leading positive pathways. Botanically, only Legumes Cover (%) maintained a significant direct link to Mn accumulation (β = 8.312, *p* < 0.001).

Iron Pathway (R^2^ = 0.150): The structural model for Iron confirmed a complete decoupling from sward botanical attributes, with all functional group coverages and richness metrics falling into non-significant pathways (*p* > 0.05). Forage Fe was directly driven by gross mineral assembly via Total Forage Ash (%), which exerted a massive positive path coefficient (β = 57.571, *p* < 0.001). Conversely, Crude Protein (%) operated as a significant negative causal pathway (β = −11.428, *p* = 0.027), confirming a direct physiological dilution process where higher nitrogen concentrations compress gross iron accumulation per unit of dry matter.

## 3. Discussion

### 3.1. Baseline Nutritional and Mineral Diagnostics: Deficiencies and Antagonistic Risks

The baseline multi-trait characterization of the examined Pyrenean hay meadows unzipped a highly distinct agronomic profile, emphasizing a clear structural decoupling between macro-nutritional templates and trace element densities. The significantly lower total forage ash content (7.14% ± 1.46%) observed in our mountain dataset relative to standard European lowland baselines (8.69–11.91%; Ref. [32]) underpins the ecological signature of extensive, low-input mountain pastoral systems. In these high-altitude environments, reduced organic and inorganic fertilization regimes restrict the luxuriance of mineral uptake, generating a thinned macro-mineral matrix that simultaneously hosts an exceptionally rich botanical sanctuary (averaging 33.31 species per plot).

However, from a livestock-nutrition standpoint, this high plant diversity does not automatically guarantee full trace element self-sufficiency for grazing or winter-fed ruminants. Our diagnostic baseline confirmed that both Manganese (47.54 ± 39.20 mg kg^−1^ DM) and Zinc (22.44 ± 9.27 mg kg^−1^ DM) mean concentrations sat systematically below the standard critical nutritional thresholds widely prescribed in international literature (60–120 mg kg^−1^ for Mn and 25–45 mg kg^−1^ for Zn; Refs. [38,39]). This systematic deficit signals a widespread, latent state of sub-optimal dietary supply across the Aragonese Pyrenees. If livestock consume this native mountain as their fodder source during winter housing, it could potentially impair structural metabolic paths, epithelial integrity, and overall reproductive efficiency in extensive cattle and sheep herds [40,41]. Conversely, Copper concentrations (10.64 ± 19.28 mg kg^−1^ DM) appeared relatively stable and adequate compared to global reference baselines (6–9 mg kg^−1^; Ref. [7]).

The most structurally alarming diagnostic feature was the high variability and extreme distribution boundaries verified for Iron (154.25 ± 181.14 mg kg^−1^ DM). Such dramatic standard deviations, frequently bursting past the upper safety thresholds of animal requirements (250 mg kg^−1^), cannot be attributed solely to internal plant translocation loops. Instead, they strongly point to external environmental driving forces, such as soil splash onto lower canopy leaves during heavy mountain rain events or atmospheric dust deposition on the sward face [4,5].

This hyper-accumulation of Fe introduces a critical clinical risk regarding microelement bioavailability. In ruminant digestive physiology, excessive dietary iron undergoes complex intraluminal interactions, acting as a powerful geochemical antagonist that directly competes for divalent metal transporter-1 (DMT1) sites in the intestinal mucosa [42,43]. Consequently, high Fe intakes actively block the metabolic absorption paths of Copper and Zinc [4,13]. Our findings prove that the trace mineral challenge in Pyrenean livestock systems is double-edged: animals must simultaneously cope with a structural dietary insufficiency of Mn and Zn, and an induced, secondary subclinical sub-optimal dietary supply of Cu and Zn triggered by the antagonistic pressure of soil-derived Iron.

### 3.2. The Role of Diversity in Mineral Accumulation

The positive trajectories links between sward taxonomic complexity and the concentrations of Zinc (Zn) and Copper (Cu) provide compelling empirical validation for the Biodiversity–Ecosystem Functioning (BEF) framework applied to multi-species forage production [12,14]. Our bivariate metrics (Table 2) showed highly significant positive correlations between species richness and both divalent cations, matching the macro-trends reported across temperate European semi-natural pastures [16,21]. Under the low-input management dynamics characterising Pyrenean hay meadows, species-rich plant communities trigger a collective biofortification phenomenon driven by niche complementarity pathways, such as vertical partitioning of root architectures across the soil profile and divergent seasonal nutrient uptake kinetics [14,15].

However, our integrated multi-tiered strategy demonstrates that taxonomic diversity operates primarily as an overarching ecological vehicle, whereas the true biochemical machinery driving Zn and Cu enrichment is heavily governed by functional group identity. Specifically, the non-leguminous forbs cluster Other Families emerged as the most dominant biological key contributor regulating these two elements. Fisiologically, dicotyledonous from families such as Plantaginaceae, Asteraceae, or Apiaceae, highly abundant across the sampled Pyrenean meadows, possess significantly higher baseline requirements and higher transport-channel capacities for divalent heavy metal cations than monocotyledonous [13,17]. This is thoroughly confirmed by our multivariate structural path models (Table 4), where Other Families cover exerted a massive, uncoupled direct causal effect on Zn (β = 18.421, *p* < 0.001) and Cu (β = 9.541, *p* < 0.001), completely overriding standard macroclimatic variations or biomass dilution patterns.

A finding of immense practical and agronomic value for mountain livestock farm viability is the “saturation threshold” unzipped via our categorical abundance tertile partitions (Figure 2). Forage Zinc concentrations did not display a linear increase along the forb abundance spectrum; instead, a sharp threshold was validated where the Medium and High tertiles achieved identical statistical separation over the Low tier. This non-linear behaviour suggests that farmers do not need to manage fields towards a complete dominance of dicotyledonous—which could potentially undermine overall dry matter yield or meadow persistence. Conversely, maintaining a balanced, moderate visual coverage of companion non-leguminous forbs (~20% to 25% cover, matching our Medium abundance tier) is sufficient to effectively trigger the mineral biofortification of the hay, elevating Zn and Cu supplies closer to the systemic animal maintenance demands without disrupting standard agronomic yield balances.

### 3.3. The Dilution Effect in Grass-Dominated Swards

The negative associations validated between Grass Cover (%) and the concentrations of essential divalent micronutrients (Zn and Cu) provide strong evidence for the phenotypic “dilution effect” within semi-natural production systems [20,44,45]. In our unsupervised global PCA space (Figure 4), the horizontal axis (PC1) revealed a sharp structural trade-off, setting dry matter yield and grass dominance in direct geometric opposition to nutritional quality traits (crude protein and RFV) and micronutrient densities. Agronomically, when mountain meadows undergo intensification or management paths that favour high grass cover (such as selective nitrogen fertilization or over-sowing), the rate of carbohydrate synthesis and structural biomass accumulation rapidly outpaces the root absorption and internal translocation rates of trace elements [46,47].

This biological mechanism is further clarified by the non-linear thresholds unzipped in our categorical tertile partitions (Figure 1 and Figure 2). While increasing Grasses Cover was associated with a compression of forage Zn and Cu baselines, it simultaneously corresponded to an upward shift in the general mineral template (Total Forage Ash), maximizing within the highest grass abundance tertile. Although silicon (SiO_2_) was not directly quantified in this study, this apparent trend is hypothesized to align with well-documented literature describing the high intrinsic capacity of the Poaceae family to actively accumulate structural silicon within their leaf epidermal cells and cell-wall matrices, an evolutionary mechanism that enhances mechanical tissue strength and reduces herbivory pressure in cold environments [48,49,50,51].

Consequently, the high total ash percentage verified in grass-dominated Pyrenean swards is mechanically “misleading” from a livestock-welfare perspective. This gross ash matrix is structurally dominated by non-digestible, insoluble mineral sheets (silicate complexes) that offer zero nutritional benefit to the animal. Concurrently, these grass-dominated tissues suffer from a severe dilution of metabolic minerals, exhibiting significantly lower densities of protein-linked cations like Zinc and Copper compared to the dicotyledonous-rich niches. Management practices that inadvertently shift the sward towards extreme grass dominance compromise the structural mineral self-sufficiency of the forage, creating a sharp trade-off where biomass yield gains are paid for with a depleted trace element profile in the final winter hay.

### 3.4. Independence of Manganese: Temporal, Climatic, and Rhizospheric Drivers

The multi-tiered analytical workflow unzipped a fascinating biochemical autonomy for Manganese (Mn) dynamics, setting it completely apart from the regulatory loops governing the other divalent cations. In our unsupervised multivariate space (Figure 4), the vertical axis (PC2) functioned as an isolated temporal and agro-climatic corridor, where the Mn vector displayed an acute alignment with Day of the Year (DOY) and Growing Degree-Days (GDD), remaining perpendicular (orthogonal) to the quality-productivity trade-offs of PC1. This complete independence from the general macro-mineral template was further confirmed by its near-zero correlation with Total Forage Ash (Table 2) and its flat causal pathway within the structural network (β = −0.214, *p* > 0.05). Such behaviours prove that Mn accumulation does not follow passive transpirational flow patterns or mechanical integration into the cell-wall silicate matrix, operating instead under strict ecophysiological and phenological scheduling [52,53,54].

Physiologically, the robust positive linear relationship validated between Mn concentration and temporal progress (DOY/GDD) reflects a cumulative senescence-driven trapping mechanism. Within the plant tissue, Manganese is an essential component of the oxygen-evolving complex in photosystem II, embedding itself heavily within the chloroplast structures [55]. Unlike highly mobile elements, structural Mn exhibits exceptionally low phloem mobility; once it is loaded into the leaf blades during early vegetative growth, it remains permanently locked in those tissues [56]. As the harvesting window is delayed across the Pyrenean cutting season (moving towards higher GDD and advanced calendar dates), the continuous transpirational influx coupled with progressive leaf aging triggers a steady, linear bio-concentration of Mn per unit of dry matter, effectively bucking the standard maturity-driven dilution observed for crude protein or total ash.

Beyond this macro-climatic timeline, our structural equation pathways (Table 3) and categorical partitions (Figure 1) revealed that Legumes Cover (%) functions as the main significant biological driver optimizing Mn densities (β = 8.312, *p* < 0.001). This specific target synergy is closely linked to rhizospheric chemical modifications characteristic of nitrogen-fixing species [57]. While rhizospheric pH and extractable soil Mn were not directly measured in this study, this legume-mediated Mn enrichment is hypothesized to stem from root-zone chemical modifications characteristic of nitrogen-fixing species. As documented in the literature, legumes actively absorb higher balances of cations over anions during symbiotic N_2_ fixation, releasing protons H^+^ into the rhizosphere [58,59]. This localized acidification can dynamically enhance the solubilization of manganese oxides into bioavailable Mn^2+^. Future studies evaluating micro-rhizospheric pH and soil-extractable fractions are recommended to empirically confirm this proposed mechanism in mountain meadows.

### 3.5. The Anomaly of Iron: Soil-Matrix Coupling, Protein Dilution, and Environmental Masking

The strong, exclusive relationship between ash content and Fe concentrations strongly points to exogenous soil contamination—such as soil splash during rainfall events or dust deposition during mechanical harvesting—rather than endogenous physiological plant uptake [60,61]. Soil typically contains iron concentrations several orders of magnitude higher than plant tissues [61]. Consequently, even minor soil adhesion significantly inflates both total ash and Fe values in the forage matrix. This processes carries critical nutritional implications: while this ‘dirt-derived’ iron increases the total analytical Fe profile of the hay, it is mostly insoluble and contributes heavily to intraluminal antagonism, severely impairing the metabolic availability of essential elements like Copper and Zinc for grazing livestock [37,43].

The clinical and statistical behaviour of Iron (Fe) across the examined Pyrenean ecosystem represented a complete departure from the biological rules governing the other divalent micronutrients. While Zn, Cu, and Mn showcased tight co-evolutionary and non-linear dependencies with sward richness and functional group identities, forage Fe concentrations remained completely flat, overlapping, and structurally homogeneous across all botanical abundance tertiles (*p* > 0.05; Figure 3). This systemic independence from the meadow’s biological configurations suggests that internal plant-mediated metabolic accumulation or functional niche differentiation plays a secondary role in regulating gross iron profiles in mountain [62,63].

Instead, our multivariate structural path models (Table 3) provided definitive mathematical evidence regarding the true driving mechanism behind Fe concentrations, unzipping a dominant, highly significant positive direct causal pathway linked to Total Forage Ash (%) (β = 57.571, t = 4.693, *p* < 0.001). Physiologically and geochemically, this massive coefficient indicates that forage iron is tightly coupled with the general inorganic mineral template. In temperate mountain grasslands, high soil iron pools—coupled with frequent summer storm events and extensive livestock movement—mechanically trigger substantial soil splash and dust deposition onto the lower canopy leaves [64,65]. Because iron is highly abundant in the soil matrix compared to plant tissues, even microscopic quantities of external soil particles adhering to the forage surface heavily distort the total elemental analysis in the laboratory, simultaneously forcing both total ash percentages and gross Fe readings upward [65]. This external environmental masking effectively creates the extreme variances and massive standard deviations observed in our baseline diagnostic table (154.25 ± 181.14 mg kg^−1^ DM).

Interestingly, the structural causal network also unravelled a significant negative direct pathway operating between Crude Protein (%) and Fe concentration (β = −11.428, t = −2.231, *p* = 0.027). This negative link validates a strict physiological dilution process [66,67]. When management or local microclimatic conditions favour early harvests or higher metabolic activity, the rapid expansion of cellular nitrogenous compounds (amino acids and proteins) outpaces the passive accumulation of both structural ash and surface-adhered soil residues [67]. Consequently, nitrogen-dense vegetative tissues structurally compress the final iron density per unit of dry matter.

Our integrated analysis proves that Iron dynamics in Pyrenean hay meadows are not regulated by the “botanical biofortification” key contributor that influences Zinc or Copper; rather, Fe behaves as a geochemically driven element, governed by the mechanical interaction between soil adherence (gross ash matrix) and the physiological expansion of vegetative proteins.

While soil geochemical pools inherently establish baseline trace element availability, general edaphic conditions across these Pyrenean meadows were relatively homogeneous (neutral-alkaline pH, high organic matter). In this context, total forage ash provided a reliable functional proxy for exogenous soil contamination, effectively isolating soil splash effects on Fe. Crucially, the strong direct structural pathways linking non-leguminous forbs to Zn (R^2^ = 0.550) and Cu (R^2^ = 0.428) demonstrate that botanical functional composition exerts a dominant regulatory control over forage micronutrient profiles, superseding background soil variability across these semi-natural swards.

## 4. Materials and Methods

### 4.1. Study Area and Experimental Design

The study was conducted in the Aragonese Pyrenees (NE Spain) over three consecutive growing seasons (2022, 2023, and 2024). Twelve representative semi-natural hay meadows were selected across a prominent altitudinal gradient ranging from 602 to 1612 mm. The sites encompass a climate gradient transitioning from Sub-Mediterranean to Mountain-Oceanic influences, with mean annual temperatures of 6.7–13.1 °C and annual precipitation varying between 718 and 1648 mm. Soils across all experimental plots are Haplic Regosol, characterized by neutral to slightly acid pH (6.8–7.8) and organic matter content between 3.6% and 9.9%.

Management intensity across the meadows reflects regional extensive livestock practices along a gradient of traditional agricultural use. Across all experimental sites (Figure 5), sward management consists of a single or double annual hay-cutting regime followed by autumn aftermath grazing. In total, 212 forage samples (n = 212) were gathered across sites, study years, and harvest treatments.

To evaluate the influence of phenological stage on mineral concentrations, forage sampling was performed at two distinct harvest times: (i) the farmer’s conventional mowing date, and (ii) an earlier cut corresponding to the flowering stage (anthesis) of the dominant grasses.

### 4.2. Botanical Characterisation

Botanical composition and community diversity were assessed at peak flowering in representative 10 m × 10 m macro-plots (100 m^2^) established within each meadow using the Braun-Blanquet cover-abundance scale. Total species richness (S, species number per 100 m^2^) and absolute percentage cover per species were recorded. For functional analysis, botanical families were aggregated into three groups: Grasses (Poaceae), Legumes (Fabaceae), and Non-Leguminous Forbs. Swards were dominated by perennial grass species (*Arrhenatherum elatius*, *Dactylis glomerata*, *Festuca rubra*, *Agrostis capillaris*), companion legumes (*Trifolium pratense*, *Trifolium repens*, *Lotus corniculatus*), and diverse non-leguminous forbs (*Taraxacum officinale*, *Plantago lanceolata*, *Sanguisorba minor*, *Knautia arvensis*). Botanical composition remained functionally stable across the three experimental years, reflecting the resilient perennial vegetation structure of these semi-natural communities.

To resolve the spatial scale relationship between the 100 m^2^ botanical plot and local biomass sampling, six temporary enclosures (40 cm × 60 cm, total area 1.44 m^2^) were randomly distributed within each macro-plot prior to vegetative growth. The 100 m^2^ spatial frame captures full plant community species richness and overarching canopy architecture, whereas the six pooled enclosures minimize micro-topographical yield bias and provide a representative, homogeneous harvest of standing herbage biomass. Temporal lags during harvest were eliminated by synchronizing cutting largely to the phenological stage of grass anthesis across the altitudinal gradient, adjusting harvest dates dynamically from late May at lower elevations to early July at higher altitudes.

### 4.3. Herbage Sampling and Sample Preparation

Standing herbage biomass was harvested by clipping vegetation inside the six plot enclosures per site at 5 cm above ground level. Total fresh weight was recorded immediately in the field using a digital scale. Representative subsamples were oven-dried at 65 °C for 48 h to constant mass to calculate forage dry matter (DM) content and scale standing herbage biomass (kg DM ha^−1^). Dried forage material was ground using an ultra-centrifugal mill (Retsch ZM 200, Retsch GmbH, Haan, Germany) equipped with a 1-mm titanium-coated ring sieve to prevent trace element contamination.

### 4.4. Forage Quality and Chemical Analysis

Forage nutritive value parameters were determined in continuous laboratory workflows. Total nitrogen was analysed via the Kjeldahl method [68] on a calibration reference subset. Crude Protein (CP, %) was calculated by multiplying total nitrogen by 6.25. High-throughput CP estimation across the complete sample set was performed using Near-Infrared Spectroscopy (NIRS DS2500, FOSS, Hillerød, Denmark) scanning monochromatic reflectance from 1100 to 2500 nm in duplicate. The NIRS calibration model (n = 150 validation subset) yielded robust predictive performance statistics: determination coefficient (R^2^ = 0.95), Standard Error of Calibration (SEC = 0.38%), Standard Error of Cross-Validation (SECV = 0.46%), and Standard Error of Prediction (SEP = 0.48%).

Total crude ash (%) was determined by incinerating ground samples in a muffle furnace at 550 °C for 5 h. Fiber fractions were analysed sequentially following the methods of Van Soest [69]. Neutral Detergent Fiber (NDF) was determined using heat-stable alfa-amylase and sodium sulphite, expressed inclusive of residual ash. Acid Detergent Fiber (ADF) was determined using cetyltrimethylammonium bromide (CTAB) in acid solution. Relative Feed Value (RFV) was computed according to [69]:

DDM=88.9−(0.779 × ADF%)

$$DMI=\frac{120}{NDF\%}$$

$$RFV=\frac{DDM\times DMI}{1.29}$$

where Digestible Dry Matter (DDM) and Dry Matter Intake (DMI) represent fibre-derived nutritional estimates.

### 4.5. Agro-Climatic Metrics and Thermal Accumulation

Agro-climatic indicators were computed from daily meteorological station data adjacent to each meadow. Harvest date was recorded as sequential Day of the Year (DOY). The onset of the active vegetative season was defined as the calendar date after 1 March on which mean daily temperature (T_mean_) exceeded 5 °C for five consecutive days. Accumulated precipitation (mm) was summed from vegetative onset to harvest. Thermal time accumulation was calculated as Growing Degree-Days (GDD) using a base threshold temperature of T_base_ = 5 °C [70]:

$$GDD=\sum \left(T_{mean}-T_{base}\right)$$

### 4.6. Trace Mineral Determination (ICP-OES)

Trace mineral concentrations: iron (Fe), copper (Cu), manganese (Mn), and zinc (Zn); were determined using Inductively Coupled Plasma Optical Emission Spectrometry (ICP-OES, Varian Vista-MPX, Palo Alto, CA, USA). Samples (0.5 g) underwent microwave-assisted acid digestion in sealed fluorocarbon vessels using 7 mL of 65% HNO_3_ and 2 mL of 30% H_2_O_2_ (Ethos One, Milestone, Sorisole, Italy). Digestates were diluted with ultrapure water to 50 mL. Analytical accuracy was confirmed using certified reference material (NIST SRM 1573a Tomato Leaves; recoveries ranged between 94% and 103% for all elements) and procedural blanks analysed every 10 samples. Analytical determination was run in triplicate per sample, with final mineral concentrations reported on a dry matter basis (mg kg^−1^ DM).

### 4.7. Statistical Analysis

All statistical analyses and data visualisation were performed in JupyterLab (3.6.3) using the Python programming language (3.13.18) [32,33]. The core analytical workflow relied on pandas (2.2.3) and NumPy (2.1.3) for data manipulation, SciPy (1.16.3) and scikit-posthocs (0.17.1) for inferential statistics, and Seaborn (0.13.2) and Matplotlib (3.10.0) for graphical representation. The dataset was first screened for normality and homoscedasticity using the Shapiro–Wilk and Levene’s tests, respectively. Given the non-normal distribution of the underlying mineral and agronomic data, a non-parametric inference framework was combined with multivariate ordination and causal modeling:

- Correlation analysis: The monotonic relationship between botanical diversity (species richness and functional group cover) and mineral concentrations was assessed using Spearman’s rank correlation coefficient (ρ).
- Categorical grouping and threshold detection: Continuous sward variables (species richness and functional group coverages) were partitioned into abundance tertiles (Low (0–33%), Medium (33–66%), and High (66–100%)). Differences in feed traits and mineral contents across tertiles were assessed via the Kruskal–Wallis H-test. Significant global discrepancies (*p* < 0.05) were evaluated using Dunn’s post-hoc test adjusted with the Holm–Bonferroni correction for multi-comparison control.
- Multivariate Ordination (PCA): To explore the unsupervised structural patterns of the dataset, a Principal Component Analysis (PCA) was performed. Prior to ordination, all continuous variables were standardized (z-score transformation) to eliminate scale bias. The selection of retained principal components was based on Kaiser’s criterion (eigenvalues > 1.0) and cumulative variance.
- Structural Causal Pathway Analysis: To disentangle the direct and indirect network of influences regulating trace element concentrations (the baseline pathways of structural equation modeling), multivariate ordinary least squares (OLS) linear regressions were executed. Predictors were screened for multicollinearity, and significant path coefficients (β) were utilized to construct directional path configurations defining direct and indirect controls over micronutrient concentrations.

## 5. Conclusions

This study highlights the key role of sward botanical composition and phenology in shaping forage trace mineral concentrations across Pyrenean hay meadows. Non-leguminous forb cover emerged as a prominent functional group associated with elevated Zn and Cu concentrations, exhibiting non-linear patterns where moderate coverage (~20–25%) corresponds to improved mineral levels without compromising overall yield. Conversely, high grass dominance was associated with an upward shift in total forage ash alongside lower Zn, Cu, and Crude Protein concentrations. Manganese concentrations were primarily linked to phenological progression (Growing Degree-Days and Day of the Year) and legume presence, whereas Iron concentrations were tightly co-varied with total ash, supporting the hypothesis of exogenous soil splash adherence.

Overall, maintaining moderate forb cover represents a practical management approach to enhance forage nutritional quality in semi-natural grasslands. However, because observed trace mineral concentrations frequently remain below recommended dietary requirements for livestock, promoting botanical diversity should be considered a complementary management tool to reduce dietary shortfalls rather than a standalone replacement for strategic mineral supplementation.

## Figures and Tables

**Figure 1:**
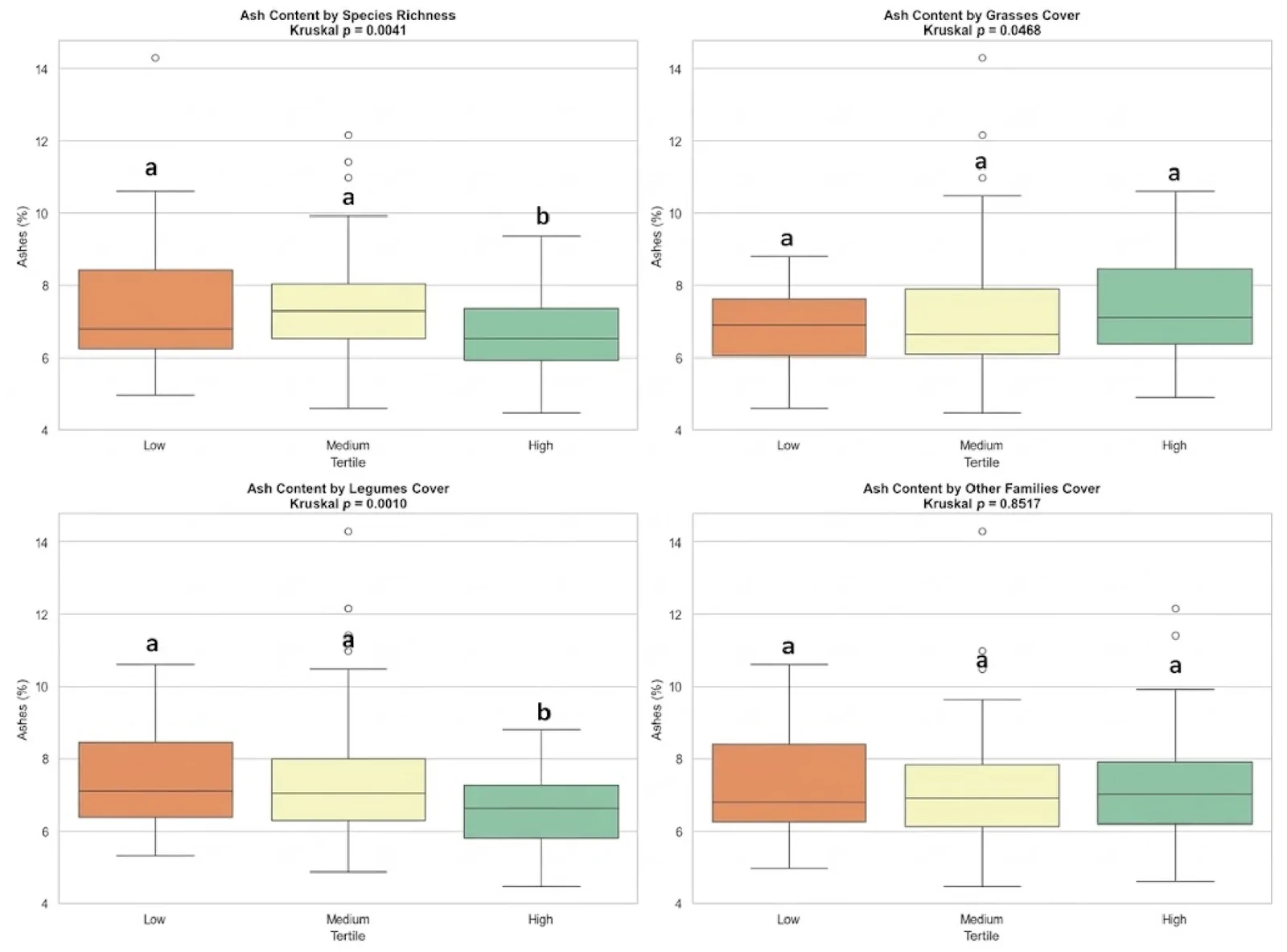
Forage total ash content (%) across abundance tertiles (Low, Medium, and High) of sward botanical variables: species richness, grass cover (%), legume cover (%), and non-leguminous forb cover (%). Boxplots display the interquartile range (IQR, 25th to 75th percentiles; box bounds), the central horizontal line represents the median, and open white circles indicate the outliers. Whiskers extend to 1.5 × IQR. Distinct lowercase letters above the boxes denote statistically significant pairwise differences between tertiles according to Dunn’s post-hoc test (*p* < 0.05). The *p*-values displayed beneath each subplot correspond to the non-parametric global Kruskal–Wallis test.

**Figure 2:**
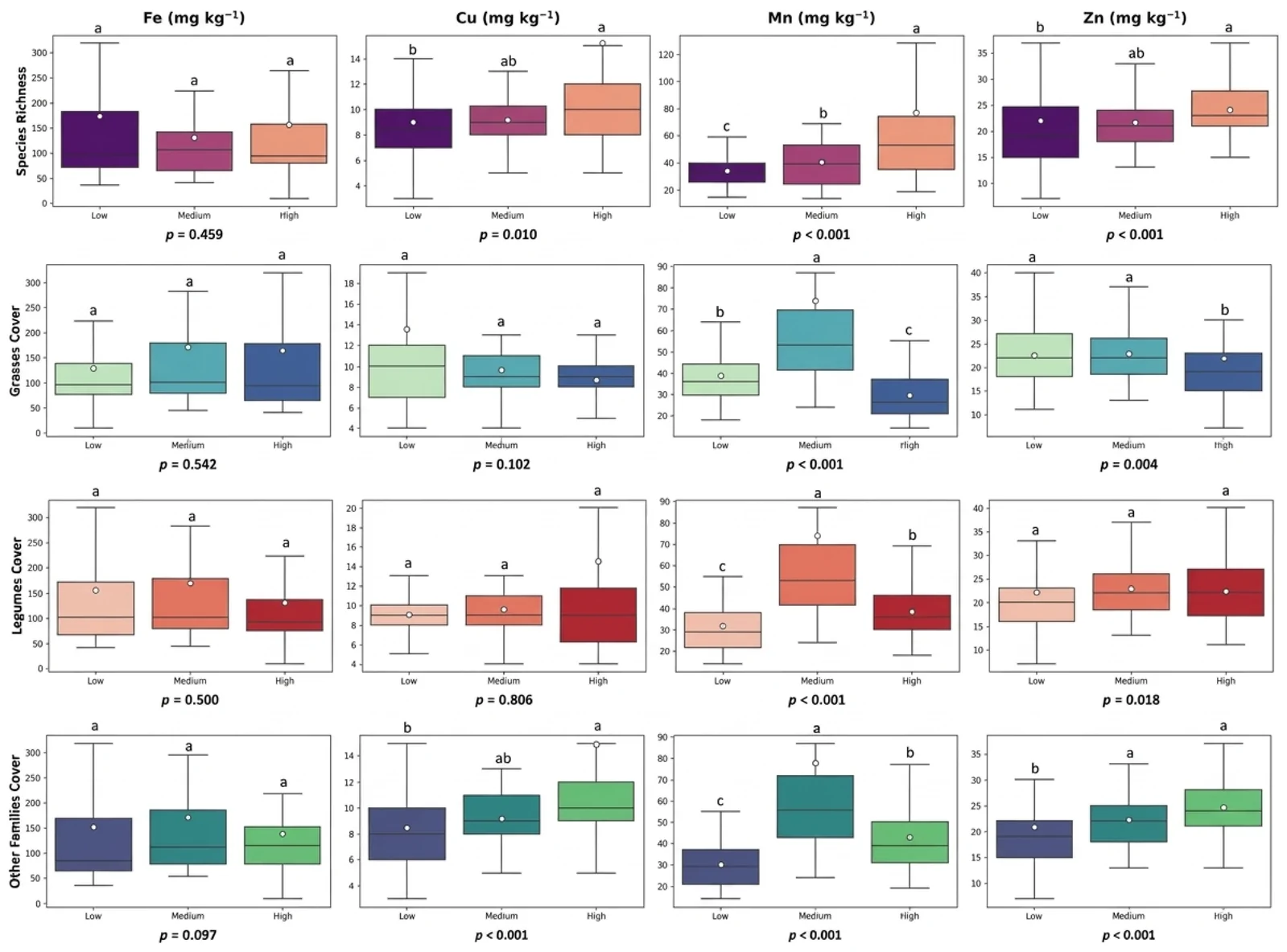
Forage trace mineral concentrations (mg kg^−1^ DM) of iron (Fe), copper (Cu), manganese (Mn), and zinc (Zn) across abundance tertiles (Low, Medium, and High) of sward botanical variables: species richness, grass cover (%), legume cover (%), and non-leguminous forb cover (%). Boxplots display the interquartile range (IQR, 25th to 75th percentiles; box bounds), the central horizontal line represents the median, and open white circles indicate the outliers. Whiskers extend to 1.5 × IQR. Distinct lowercase letters above the boxes denote statistically significant pairwise differences between tertiles according to Dunn’s post-hoc test (*p* < 0.05). The *p*-values displayed beneath each subplot correspond to the non-parametric global Kruskal–Wallis test.

**Figure 3:**
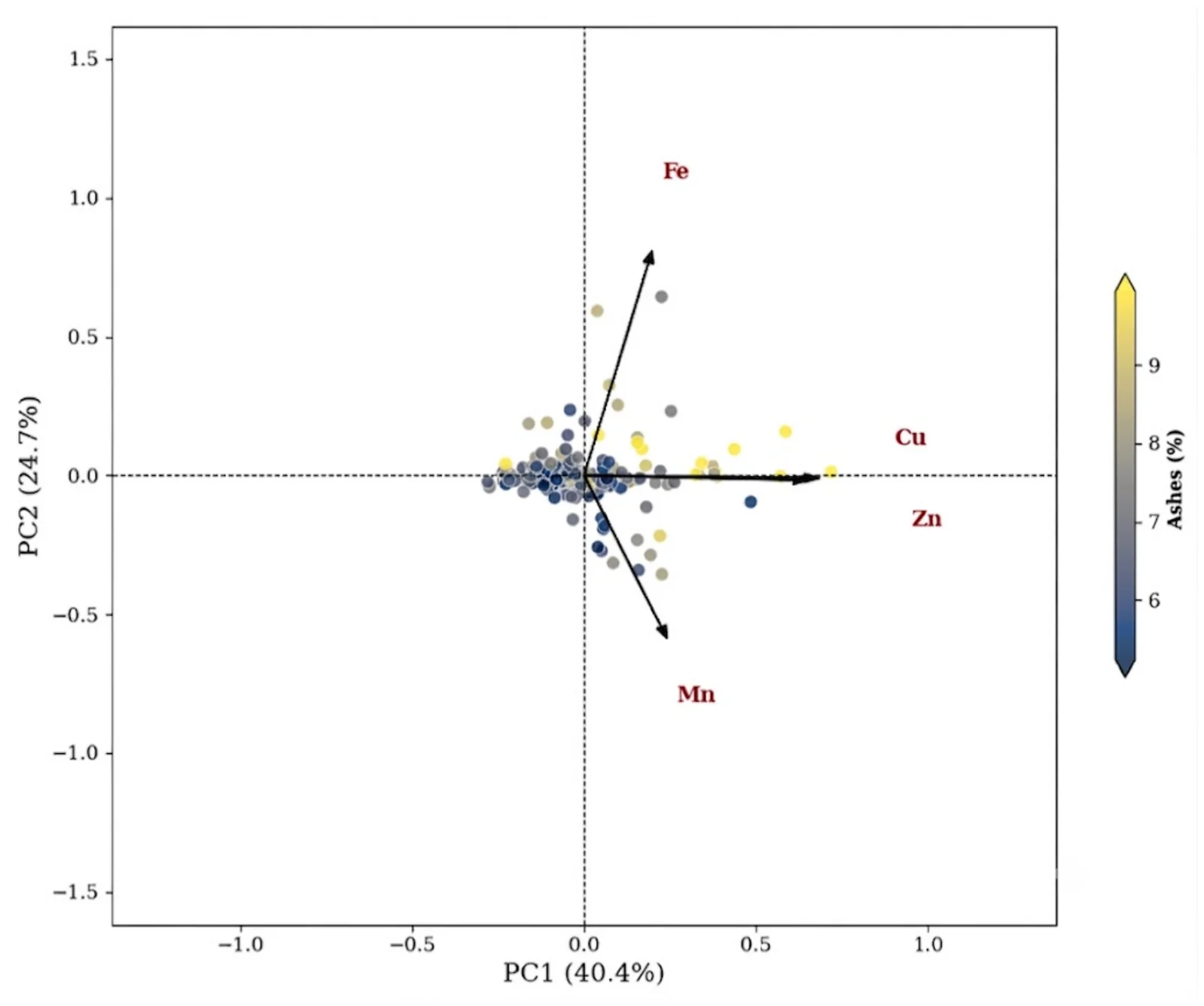
Principal Component Analysis (PCA) biplot illustrating the covariance structure of micronutrients (Fe, Cu, Mn, and Zn) in forage samples. Vectors represent the variable loadings, with direction and length indicating the correlation between nutrients and their contribution to the components. Sample points are coloured by total ash content to highlight the relationship between specific trace elements and the total mineral fraction.

**Figure 4:**
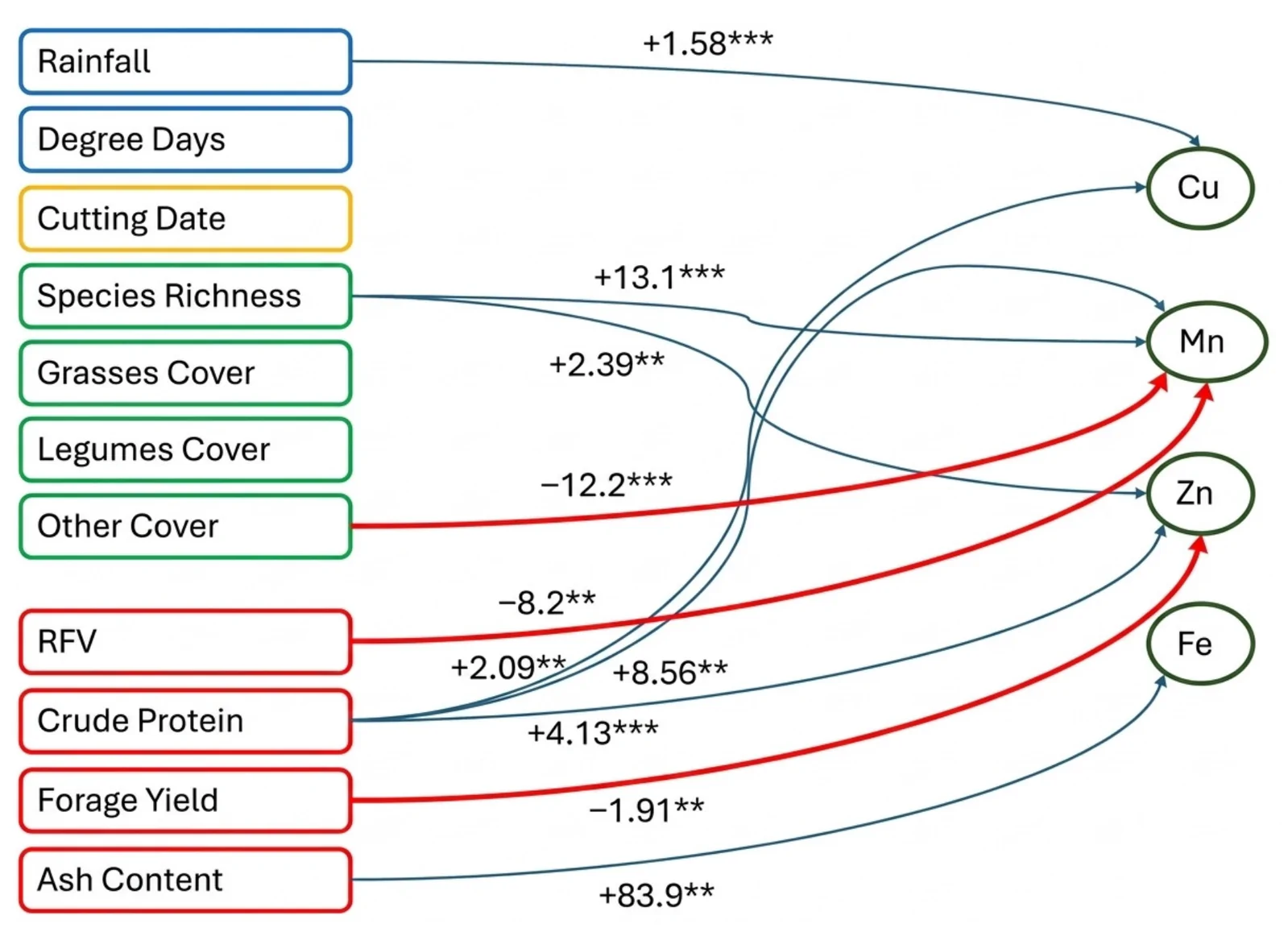
Global structural path diagram illustrating the hierarchical relationships between environmental drivers, forage quality mediators, and micronutrient outcomes. Values on paths represent standardized coefficients. Significance levels: ** *p* < 0.01, *** *p* < 0.001. Solid lines indicate positive influences; dashed lines indicate negative influences.

**Figure 5:**
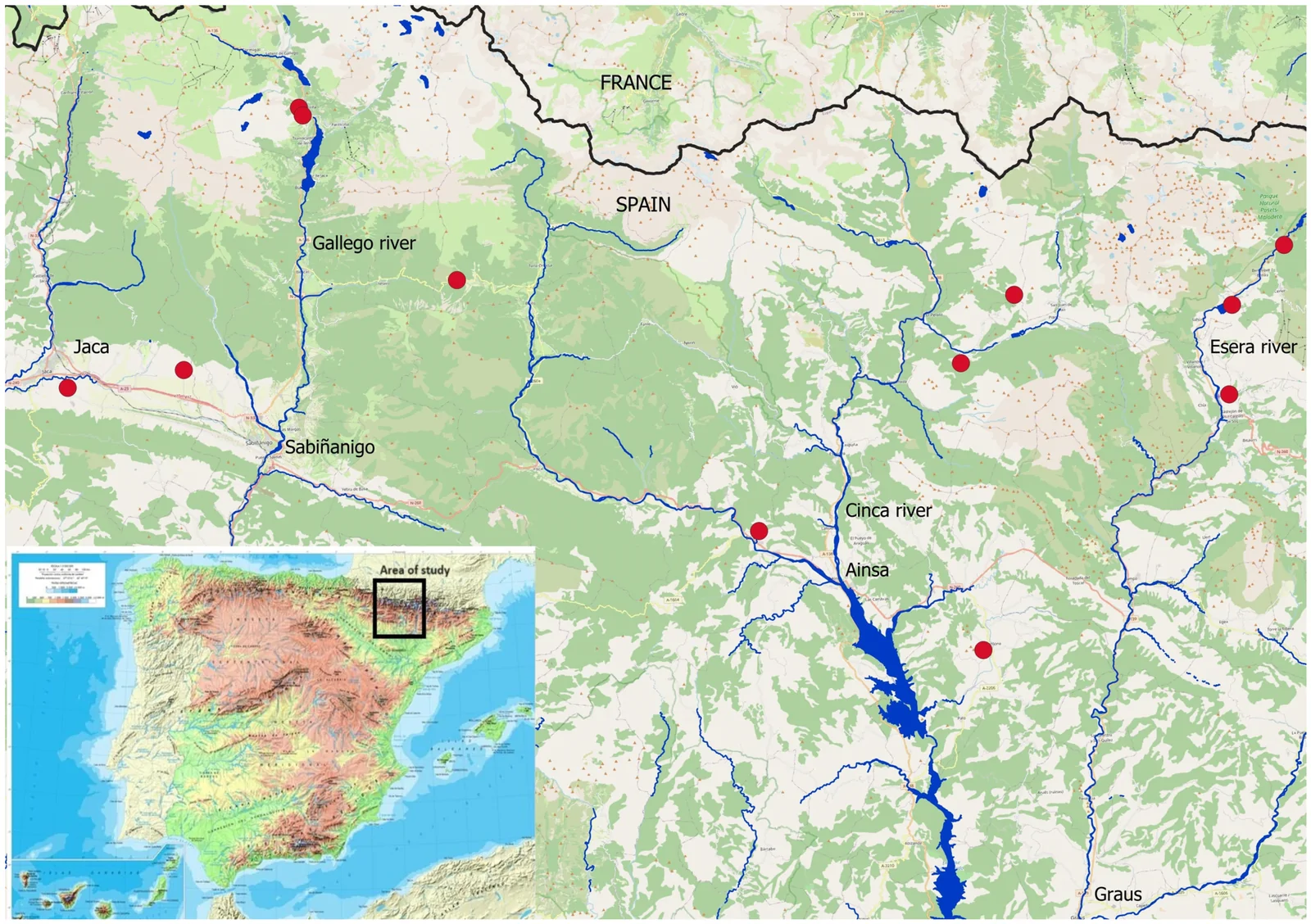
Geographical distribution of the 12 selected semi-natural hay meadows across the altitudinal and management gradients in the Central Pyrenees (Aragon, NE Spain). Red circles indicate the exact locations of the experimental sampling plots. The bottom-left inset map displays the regional positioning of the study area within the Iberian Peninsula.

**Table 1:** Baseline descriptive statistics of sward architecture, general forage quality parameters, and trace mineral concentrations in Pyrenean hay meadows (n = 212) compared with standard international literature ranges.

	Study	References
Species richness (n)	33.31 ± 14.67	15–51 [3]
Grass cover (%)	49.13 ± 22.14	16.3–80.3 [3]
Legume cover (%)	24.69 ± 15.27	0.1–45.0 [3]
Other families cover (%)	25.48 ± 15.72	4.1–68.7 [3]
Ashes (%)	7.14 ± 1.46	8.69–11.91 [36]
Fe (mg kg^−1^)	154.25 ± 181.14	100–250 [7,8]
Cu (mg kg^−1^)	10.64 ± 19.28	6–9 [7,25]
Mn (mg kg^−1^)	47.54 ± 39.20	60–120 [7,11]
Zn (mg kg^−1^)	22.44 ± 9.27	25–45 [7,37]
Cutting date (day of year)	155.32 ± 16.67	140–171 [5]
Yield (kg ha^−1^)	4582.13 ± 2540.44	2042–7122 [3]
Crude Protein (%)	12.40 ± 3.83	8.7–13.5 [5]
RFV	118.38 ± 28.68	70–86 [5]
Rain (mm)	386.37 ± 201.91	223–355 [5]
Degrees Days (Cº day)	1109.16 ± 298.34	982–1446 [5]

**Table 2:** Spearman’s rank correlation coefficients (ρ) between botanical diversity variables and forage mineral concentrations. Asterisks indicate statistical significance: * *p* < 0.05, ** *p* < 0.01, *** *p* < 0.001. Non-significant correlations are displayed without asterisks.

	Fe	Cu	Mn	Zn
Species richness	0.03	0.23 ***	0.39 ***	0.34 ***
Grass cover	0.03	−0.14 *	−0.12	−0.20 **
Legume cover	−0.04	0.03	0.33 ***	0.11
Other families cover	0.11	0.29 ***	0.25 ***	0.40 ***

**Table 3:** Factor loadings and explained variance for the first four principal components of the micronutrient analysis. Values represent the correlation between original variables and each principal component.

	PC1 (40.4%)	PC2 (24.7%)
Fe	0.198	0.809
Cu	0.656	−0.018
Mn	0.242	−0.587
Zn	0.687	−0.010

**Table 4:** Multiple linear regression pathway models defining the direct drivers and standardized coefficients (β) for Zinc, Copper, Manganese, and Iron concentrations in Pyrenean mountain meadows (n = 212). Model F-statistics and collective covariance matrices match regional baselines. Significance levels for specific pathways: * *p* < 0.05, ** *p* < 0.01, *** *p* < 0.001.

Target Micronutrient	Predictor Variable	Pathway Coefficient (β)	Standard Error (SE)	t-Value	*p*-Value
Zinc (*Model R*^2^ = 0.550)	Intercept	−0.021	0.041	−0.512	0.609
	Other Families Cover (%)	18.421	2.114	8.713	<0.001 ***
	Total Forage Ash (%)	14.215	3.012	4.719	<0.001 ***
	Dry Matter Yield	−0.004	0.005	−0.800	0.424
	Grass Cover (%)	−1.104	0.841	−1.312	0.191
Copper (*Model R*^2^ = 0.428)	Intercept	0.014	0.049	0.285	0.775
	Other Families Cover (%)	9.541	1.952	4.887	<0.001 ***
	Crude Protein (%)	6.412	2.104	3.047	0.003 **
	Total Forage Ash (%)	11.204	3.141	3.567	<0.001 ***
	Species Richness	1.014	0.912	1.111	0.267
Manganese (*Model R*^2^ = 0.3120)	Intercept	−0.084	0.062	−1.354	0.177
	Cutting Date (DOY)	22.145	4.102	5.398	<0.001 ***
	Growing Degree-Days	12.411	3.514	3.531	<0.001 ***
	Legumes Cover (%)	8.312	2.014	4.127	<0.001 ***
	Total Forage Ash (%)	−0.214	1.952	−0.109	0.913
Iron (*Model R*^2^ = 0.150)	Intercept	−0.051	0.058	−0.871	0.385
	Total Forage Ash (%)	57.571	12.267	4.693	<0.001 ***
	Crude Protein (%)	−11.428	5.122	−2.231	0.027 *
	Species Richness	2.028	1.522	1.332	0.184
	Dry Matter Yield	−0.007	0.007	−1.091	0.277

## Data Availability

Dataset available on request from the authors. The raw data supporting the conclusions of this article will be made available by the authors on request.

## Abbreviations

The following abbreviations are used in this manuscript:

PCA	Principal Component Analysis
SEM	Structural Equation Modelling
DM	Dry Matter
RFV	Relative Feed Value
DOY	Day Of Year
GDD	Growing Degree-Days
CP	Crude Protein
SEC	Standard Error of Calibration
SECV	Standard Error of Cross-Validation
SEP	Standard Error of Prediction
